# Myeloid Cell-Mediated Trained Innate Immunity in Mucosal AIDS Vaccine Development

**DOI:** 10.3389/fimmu.2020.00315

**Published:** 2020-02-28

**Authors:** Yongjun Sui, Jay A. Berzofsky

**Affiliations:** Vaccine Branch, National Cancer Institute, National Institutes of Health (NIH), Bethesda, MD, United States

**Keywords:** trained innate immunity, vaccinia virus, TLR ligands, IL-1, interferon, hematopoietic stem cell and progenitor cells

## Abstract

Trained innate immunity has recently emerged as a novel concept of innate immune cells, such as myeloid cells, exhibiting immune memory, and nonspecific heterologous immunity to protect against infections. The memory and specificity are mediated by epigenetic, metabolic, and functional reprogramming of the myeloid cells and myeloid progenitors (and/or NK cells) in the bone marrow and peripheral tissues such as gut and lung mucosa. A variety of agents, such as BCG, viruses, and their components, as well as TLR agonists, and cytokines have been shown to be involved in the induction of trained immunity. Since these agents have been widely used in AIDS vaccine development as antigen delivery vectors or adjuvants, myeloid cell mediated trained immunity might also play an important role in protecting against mucosal AIDS virus transmission or in control of virus replication in the major gut mucosal reservoir. Here we review the trained innate immunity induced by these vectors/adjuvants that have been used in AIDS vaccine studies and discuss their role in mucosal vaccine efficacy and possible utilization in AIDS vaccine development. Delineating the protective effect of the trained innate immunity mediated by myeloid cells will guide the design of novel AIDS vaccines.

Trained immunity is a novel concept that innate immune cells, such as monocytes/macrophages, have certain level of immune “memory” properties to respond to second stimulations. Bacterial, fungal, and viral components, as well as cytokines and TLR agonists have the potential to induce trained immunity. Innate immune cells, after stimulation with these stimulants, can display long-term changes in their functional programs. This can be achieved through epigenetic or metabolic programming of the innate cells (1, 2). When they encounter a second stimulation, the trained innate cells produced either an increased level of cytokines/chemokines, or decreased level of immune mediators, which constitutes the two opposite programs of the trained immunity: namely, training and tolerance programs. Though there are still conflicting opinions on whether these changes can be accounted as true immune “memory,” nevertheless, these changes can be maintained for a long period of time and can mediate protection or lead to auto-inflammatory diseases upon secondary stimulation. In this context, it should be noted that analogous epigenetic changes are also mediators of memory states in T lymphocytes at an individual cell level (3, 4). Thus, while innate cells do not have antigen-specific clones as do T and B cells that can undergo expansion to mediate memory at a population level, they are just as capable of epigenetic and metabolic changes at the individual cell level as lymphocytes to maintain a memory-cell state (Figure 1). Therefore, it should not be so surprising to see this type of memory state in innate cells like monocytes.

**Figure 1:**
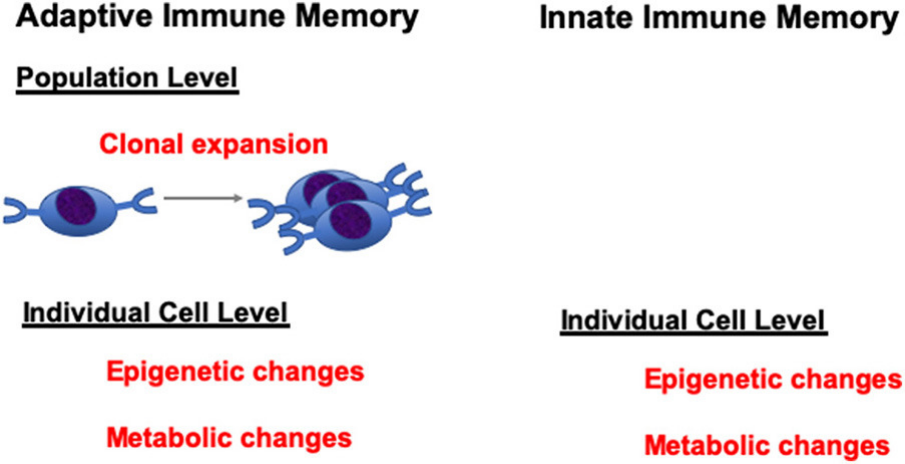
Adaptive vs. innate immune memory.

Epigenetic and metabolic pathways are the substrates of trained immunity. The fungal product β-glucan, which provided the first evidence of trained immunity in vertebrates, induced stable and genome-wide changes in histone methylation by increasing the trimethylation of H3K4me3 of many genes including TNFα and IL-6 (5). The induced epigenetic program could be maintained for a long period of time and led to enhanced gene transcription upon the second challenges. Trained immunity was also found linked to metabolic pathways. Glycolysis, glutaminolysis, and the cholesterol synthesis pathway are essential for β-glucan-induced trained immunity (6, 7). Upregulation of glycolysis is required during immune cell activation (activated T cells and proinflammatory macrophages). Though less efficient, glycolysis generates faster production of ATP, which provides energy for cell activation in a timely fashion. Furthermore, epigenetic and metabolic pathways are interconnected. After priming with β-glucan, genes that are involved in glycolysis were epigenetically upregulated (8).

Developing an HIV vaccine is the ultimate solution to curtail the HIV pandemic. However, this remains a big challenge as only one out of six phase III HIV vaccine clinical trials showed efficacy to date (9, 10). Most HIV vaccine platforms include a complex regimen of viral vectors and adjuvants, which have the potential to induce trained immunity (Figure 2). Viral vectors, such as poxviral vectors, induce trained immunity. Trained immunity memory can also be generated directly by TLR ligands, or indirectly by IL-1 (promoted by alum adjuvant) and interferons (induced by TLR ligands or produced by vaccine-activated cells). Other diet factors or microbiota can affect the trained innate immunity as well. Trained immunity is especially important and may be helpful when effective HIV vaccines are currently not available. Here, we review data on trained immunity induced by these components in myeloid cells and discuss their possible involvement in mediating the vaccine efficacy of the previous published HIV vaccine studies. We also consider the possible utilization of trained immunity in future HIV vaccine development.

**Figure 2:**
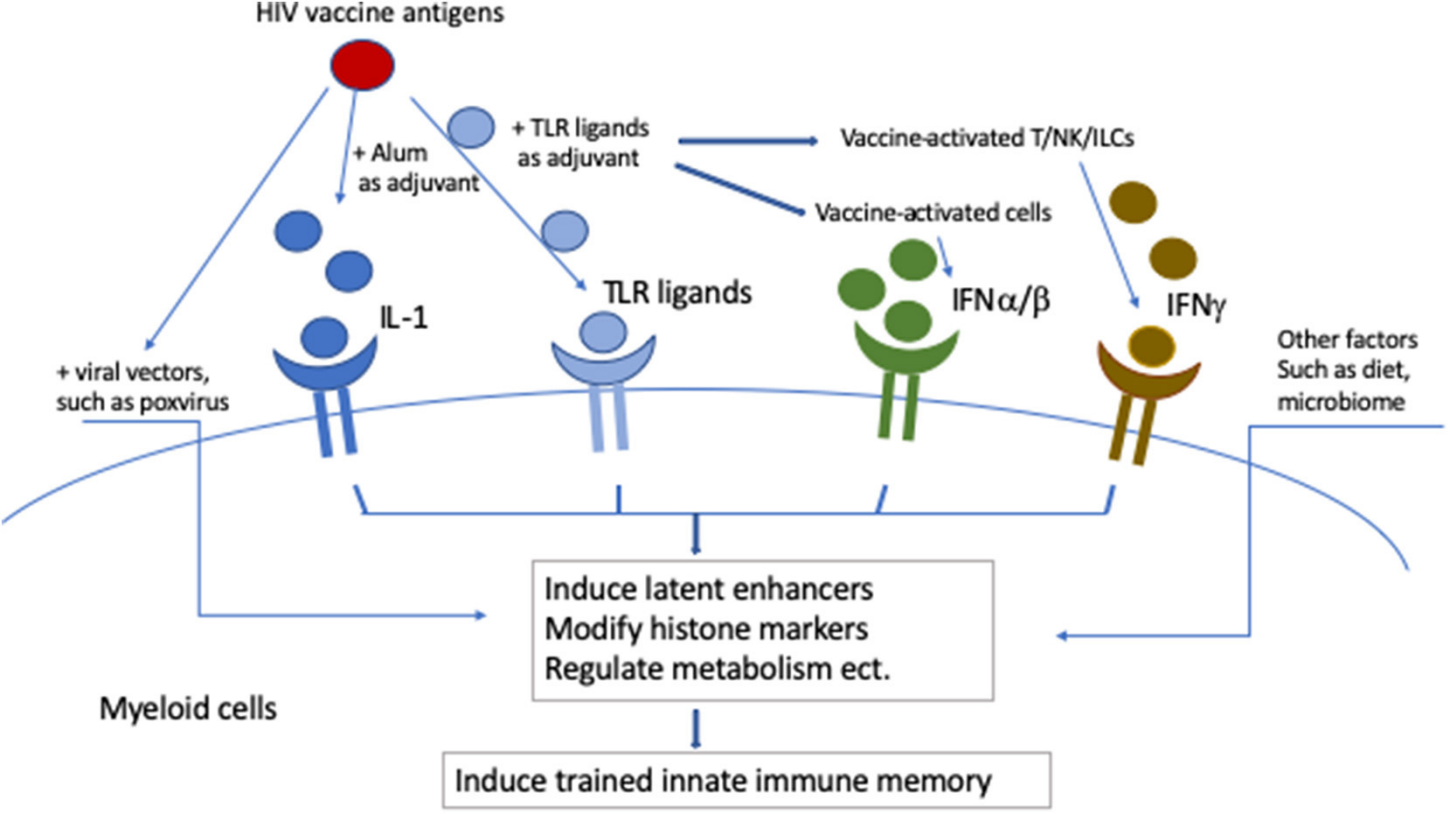
Potential induction of trained innate immunity by complex HIV vaccines. Viral vectors, such as poxviral vectors, induce trained immunity. Trained immunity memory can also be generated directly by TLR ligands, or indirectly by IL-1 (promoted by alum adjuvant) and interferons (induced by TLR ligands or produced by vaccine activated cells). Other diet factors or microbiota can affect the trained innate immunity as well.

## Poxvirus-Mediated Induction of Trained Immunity

As attenuated recombinant poxvirus vectors are safe and highly immunogenic against expressed foreign genes, they constitute excellent HIV/AIDS vaccine candidates (11). Aventis Pasteur live recombinant canarypox vector (ALVAC), modified Vaccinia Ankara (MVA), NYVAC and fowlpox are widely studied HIV poxvirus vectors (12). In the only HIV vaccine trial (RV144 trial) that showed efficacy (only 31%), four injections of recombinant HIV-ALVAC, and two injections of AIDSVAX B/E gp120 in alum were administrated (9). A later study found that the non-neutralizing IgG antibody responses against the V1V2 region of the envelope were correlated inversely with risk (13). However, several passive adoptive transfer studies with non-neutralizing anti-envelope antibodies in the macaque models failed to mediate protection (14–17). This raised the question whether there were other mechanisms involved. Trained immunity is one of the potential mechanisms.

Epidemiologic and experimental data showed that poxvirus itself could induce trained immunity. Smallpox vaccine, vaccinia, used to be a routine immunization until smallpox was eradicated in 1977. Several observational studies reported that immunization with vaccinia vaccine reduced the overall mortality in adults of Guinea-Bissau and Denmark (18–20). A sex bias was found in one of the studies with a stronger effect in females (18). Moreover, an inverse association of risk of non-Hodgkin lymphoma and melanoma was found with previous vaccinations with smallpox (21, 22). This non-specific protective effect suggested that vaccinia might induce trained immunity to mediate protection against other infectious diseases and cancers. Scherer et al. compared the gene expression pattern of human peripheral blood mononuclear cells following Aventis Pasteur smallpox vaccine (23). They found that smallpox vaccine upregulated genes associated monocytes or macrophages. A surprising number of genes, including IL-8 and IL-18, exhibited significant changes even 50–60 days post vaccination, supporting the induction of trained immunity (23). Similarly, monocyte and macrophages from vaccinia infected mice produced more TNF and IL-6 after being re-stimulated *in vitro* with HSV-infected cells, further suggesting training programs of trained immunity might be induced by vaccinia (24).

Recently, we and others found evidence that myeloid cell-mediated trained immunity might be involved in mediating protection using similar immunization protocols like RV144 in macaque models (25–27). Vaccari et al. found that hypoxia and inflammasome activation in CD14+CD16– monocytes are correlates of decreased risk of SIV acquisition after vaccination with DNA/ALVAC/gp120 platform in macaques (25, 27). We demonstrated in the macaques vaccinated with MVA/FLSC (full-length single chain recombinant gp120 fused with two domains of CD4 to maintain the CD4-induced conformation) with complex adjuvants that reduced infection risk was achieved in the absence of protective antibody responses against HIV envelope (26). The protection correlated with CD14+DR- monocytes induced by the vaccine; but not the viral-specific T cell responses induced by the vaccine. We proved that trained immunity was induced by re-exposing the monocytes *ex vivo* with challenge SHIV virus to mimic the *in vivo* scenario. The monocytes from vaccinated animals produced higher amounts of TNFα, IL-6, and MIP1α than those from the naïve animals upon re-stimulation with virus. Interestingly, the increased production of cytokines/chemokine also correlated with *in vivo* challenge outcome, suggesting that trained immunity mediated protective efficacy. Since the interval between the last boost and first challenge was 8 weeks, we believe that trained immunity was induced and mediated protection in this RV144-like trial. However, since we have included multiple components in the vaccine, in this study we cannot dissect the mechanism of induced trained immunity to attribute it to MVA or TLR 2, 3, 9 agonists, IL-15 and mLT, or the combination. Further study is required to delineate the mechanisms.

Notably, different poxvirus vectors induced different innate immune profiles, which makes the interpretation of HIV vaccine studies difficult. One study found that after administrating ALVAC, MVA, and NYVAC poxvirus vaccine vectors to macaques, ALVAC induced a very different proinflammatory cytokine/chemokine profile from MVA and NYVAC, characterized by a higher induction of proinflammatory and IFN-related antiviral cytokines and chemokines at day 1 post vaccination (28). Furthermore, the stimulatory phenotypes were all reduced when the animals were re-exposed to these poxvirus vectors (28). Previous reports found that MVA induced stronger IFN-stimulated genes, while NYVAC promoted proinflammatory genes after infection in HeLa cells (29, 30). These differences might lead to potentially different biological effects, though it remains unknown to what extent these induced innate immune profiles contributed to vaccine efficacy. Nevertheless, the different innate immune responses induced by these vectors can potentially influence adaptive immunity, as well as trained immunity. Since not all trained immunity contributes to protection, studies to identify the distinct trained innate immunity profile which contributes to HIV/SIV vaccine efficacy are needed. This will facilitate the interpretation of vaccine results, and the manipulation of the reagents to induce protective trained immunity in future HIV/AIDS vaccine development.

## Adjuvant-, Toll-Like Receptor (TLR) Agonist- and Cytokine-Mediated Induction of Trained Immunity

Both Toll-like receptor (TLR) agonists and cytokines have been widely used adjuvants in HIV/SIV vaccine development. Accumulating data from *in vivo* and *in vitro* studies support the notion that these adjuvants not only enhanced the antigen-specific T cell and B cell responses, but also induced trained immunity by imprinting the innate immune cells with epigenetic and metabolic modifications, which resulted in enhanced or decreased responses upon re-stimulation.

TLRs, type I transmembrane proteins, belonging to the pattern recognition receptor family, are expressed on the innate immune cells. Once engaged by their distinct ligands, TLRs activate innate immune cells, and participate in the initiation of adaptive immune responses (31). As adjuvants, TLR agonists enhanced the potency of vaccine-induced adaptive immunity. Ten TLRs have been identified in humans, and most of them, such as TLR, 2, 3, 4, 7, 8, and 9 agonists, have been tested as adjuvants in HIV/SIV vaccine studies (26, 32–36). In these studies, TLR agonists promoted efficient vaccine antigen delivery, induced more pronounced cell activation, and increased T follicular helper cell differentiation and germinal center formation, which led to stronger antigen-specific T cell and antibody immune responses.

Recent literature suggested that TLR agonists and cytokines also modulate the functional programming of monocytes/macrophages and render them “memory” properties for protection. One molecular mechanism of retaining the memory in myeloid cells was shown to be through the emergence of latent enhancers, which are the genomic regulatory elements unbound by transcriptional factors and unmarked in unstimulated cells (37). Upon activation with TLR agonists or cytokines, latent enhancers were induced, in addition to the activation or repression of the pre-existing poised enhancers. Interestingly, after washout of the stimuli, most of the enhancers returned to normal states, whereas a large fraction of latent enhancers remained stably epigenetically marked to keep the “memory” of the first stimulation. Specifically, selective retention of H3K4mel upon signal termination resulted in a faster and stronger responses upon re-stimulation (37). The complex repertoire of latent enhancers induced by various TLR agonists, including TLR, 2, 4, and 9, and cytokines such as IL-4, IFNγ, IL-1β, TNFα, and TGFβ have been uncovered (37). Each TLR agonist and cytokine stimulated a distinct repertoire of latent enhancers, which was considered as the epigenomic footprint of the stimulus (37).

Interestingly, the dose of the stimulation also determines the types of response. In the well-studied lipopolysaccharide (LPS) stimulation experiments, the concentration of LPS governed whether tolerance or priming of the monocytes was induced (38). LPS exposure induced several types of epigenetic modifications, including two gene-specific chromatin modifications that associated with silencing of pro-inflammatory mediators, or priming of antimicrobial effectors, which were the molecular mechanisms of tolerance and training (39). Similarly, *in vitro* exposure to other TLR agonists such as Pam3CSK4, Flagellin, polyIC, R848, and CpG, as well as NLR agonists TriDAp or MDP also altered the functional fates of monocytes and induced two opposing functional programs based on the nature and concentration of the agonists (40). High concentrations of the TLR agonists induced tolerance with the exception of CpG (for which tolerance is inherent at any dose), while low concentrations of TLR agonists generally abolished the tolerance and induced training of the monocytes. This led to a diminished or enhanced production of the cytokine/chemokine upon second stimulation (40). Mechanistically, the trained immunity in the monocytes induced by TLR agonists was dependent on histone methylation and acetylation (40).

Some viral vectors, such as vaccinia virus, have been demonstrated to be directly sensed by TLRs as well. Some reported that vaccinia virion was recognized by TLR2 (41–44), while the others found that TLR2 was not involved at all (45). Differences in effects may reflect differential pairing of TLR2 with TLR1 or TLR6. Specifically, TLR2/6 has been shown to mediate the innate immune sensing of MVA in macrophages to produce chemokines, IFNβ and IL-1β (42). In this respect, viral vectors can induce trained immunity by indirectly working through TLRs.

It worth mentioning that TLRs in collaboration with other innate receptors such as Nod-like receptors (NLRs), and C-type lectin receptors (CLRs) can shape innate/adaptive and maybe trained immunity, and some of the effects of adjuvants are not through TLRs, but through CLRs and NLRs (46–48).

Cytokines participate in the induction of trained immunity to potentially impact vaccine efficacy in several ways. First, cytokines can directly induce trained immunity. Cytokines such as TNFα, IL-4, IFNγ, or TGFβ induced latent enhancers, which were epigenetically modified after encounter with cytokines (37). Upon second stimulation, these cytokine-modified latent enhancers responded either more slowly or more quickly than the non-modified enhancers, which resulted in enhanced or decreased production of the gene products (37). Secondly, cytokines can be induced indirectly by other adjuvants. For example, alum, an adjuvant widely used in HIV and other vaccine development, induces the production of IL-1. IL-1 is one of the key components mediating the induction of trained immunity (49). As mentioned before, IL-1β induced trained immunity itself. Human monocytes treated with IL-1β *in vitro* had the potential to express high levels of TNFα and IL-6 upon re-stimulation (49, 50). The training was accomplished by the epigenetic modification of the promoter regions of TNFα, IL-6, and IL-1β (50). Furthermore, IL-1 was involved in the induction of trained immunity through modulation of metabolic pathways. Cheng et al. demonstrated that a complicated metabolic pathway shift led to the induction of trained immunity, where IL-1 and hypoxia-inducible factor 1α (HIF1α) were involved (8). Human monocytes treated with beta-glucan displayed a core metabolic shift from oxidative phosphorylation to aerobic glycolysis. This shift was mediated by the AKT/mTOR/ HIF1α pathway, and gene knock-out experiments showed that HIF1α is essential for the process. HIF1 is a heterodimeric transcription factor composed of HIF1α and HIF1β subunits, which regulate over 70 genes responding to hypoxia (51). HIF1β is constitutively expressed, while the expression of HIF1α is tightly regulated by O_2_ concentration. Since the promoter region of IL-1β contains several binding sites of HIF1α, HIF1α can directly target the IL-1β gene (52, 53). Similarly, in the LPS-induced IL-1β production, LPS-treated macrophages increased the expression levels of succinate, which acted as a metabolite in innate immune signaling, and which then stabilized HIF1α. IL-1β, as a target of HIF1α, was induced after exposure to LPS (53). On the other hand, IL-1β can induce HIF1α under normoxic conditions via NF-κB/COX2 pathway (54). Altogether, accumulating evidence showed that high IL-1β level, possibly as a inducer or as a mediator of trained immunity, plays an important role in protecting against bacteria, candida and viral infections (55–58).

Another group of cytokines, which are widely induced by most vaccines, including HIV vaccines, are interferons (IFNs). During vaccination, IFNα/β are usually induced at the early stages, while IFNγ, mainly produced by vaccine-activated T cells, NK cells, and NKT cells, is produced at the later stages. IFNs are classified into three groups, Type I (IFNα/β), II(IFNγ), and III IFNs (IFN-λ), based on the structure of their receptors. IFNs, especially IFNγ, turned out to have much broader effects on both arms of the immune system than most of the cytokines (59). They have strong antiviral activity and have been widely used to treat viral infections (60). Different IFNs, used as adjuvants, have been reported to induce distinct pathways to enhance the efficacy of vaccines (61).

Recent data suggested that exposure of monocytes/macrophages to IFNs leads to the induction of innate trained memory. IFNs induce chromatin remodeling, and lead to the demonstration of an “interferon epigenomic signature” in the treated monocyte/macrophages, which includes activation of latent enhancers, modulation of histone markers, regulation of chromatin accessibility, and alteration of enhancers and promotors (37, 62–68). For example, IFNβ induced epigenetic memory in fibroblasts and bone marrow-derived macrophages. Kamada et al. showed that IFNβ treatment led to faster and greater transcription of IFN-stimulated genes upon re-stimulation. This was achieved through accelerated recruitment of RNA polymerase II and phospho-STAT1 and coincided with histone H3.3 and H3K36 modifications. On the other hands, IFNγ was originally identified as “macrophage activating factor” and can polarize macrophages by modifying chromatin to reprogram transcriptional landscapes, which confer innate immunity to macrophages (69).

IFNs also interacted with TLR ligands to modulate immune responses. After stimulation with IFNγ and TLR ligands, human macrophages showed strong synergistic activation of inflammatory cytokine production, which was due to sustained occupancy of STAT1, IRF-1, and associated histone acetylation at promoter and enhancer regions of TNF and IL6 loci (63). IFNs have been observed to abrogate tolerance, which was induced by previously exposure to TLR ligands in macrophages (62). Furthermore, type I interferons cooperatively altered chromatin states of the macrophages with other cytokine such as TNF to induce transcriptional cascades to prevent the silencing of genes induced by TLR4 (67).

## Other Adjuvants and Vectors: Adenovirus-Vectored Vaccine and AIDS Viruses Themselves

Recombinant attenuated adenovirus vectors, described as ideal platforms for vaccine delivery vectors, have been widely used in HIV/SIV clinical and experimental vaccine development. These vectors are safe, highly immunogenic, and able to express large amounts of antigens (70). However, two phase IIb clinical trials to evaluate the Merck human adenovirus serotype-5 vector expressing HIV gag/pol/nef did not demonstrate a decrease in HIV acquisition (71–74). To make matters worse, the STEP trial showed that the vaccination was associated with enhanced susceptibility to HIV infection in uncircumcised adenovirus serotype-5 seropositive men (71). The possibility that vaccination with adenovirus vector increased mucosal T cell activation was confirmed in some of the macaque studies (75), but not in others (76). Based on the recent finding that adenovirus can also induce trained immunity, it is tempting to hypothesize that trained immunity might affect the efficacy of these clinical trials. The fact that the enhanced HIV susceptibility occurred in adenovirus serotype-5 seropositive men indicated that these persons had been exposed to adenovirus 5 before. Whether the exposures to adenovirus led to the induction of trained immunity and/or epigenetic or metabolic modifications of innate cells such as myeloid cells are open questions, and worth investigation.

In addition, we recently found that a live attenuated AIDS virus, SHIV, could protect against intrarectal challenge with SIV in the absence of anti-envelope antibodies, through a mechanism involving trained innate immunity (77). The protection was also independent of CD8 T cells induced by the vaccine, as shown by CD8 T-cell depletion studies. Rather, epigenetic changes were detected in monocytes that may mediate such trained innate immunity. Thus, even AIDS viruses themselves can induce trained innate immunity.

## Trained Immunity Memory

Trained immunity memory has been defined as increased or decreased responsiveness to a secondary stimulation by innate immune cells. The short life span of these cells challenged the notion of long-term maintenance of trained immunity memory by matured innate cells. For example, in the cases of Bacillus Calmette-Guérin (BCG) vaccination, the epigenetic signature of innate cells lasts for up to 1 year (78). Thus, immune progenitor cells must be involved. Indeed, two recent studies found that hematopoietic stem cell (HSC) and progenitor cells (HPC) in the bone marrow were modulated with altered epigenetic landscapes and transcriptional profiles, which confer the memory properties for the trained immunity (79, 80). Kaufman et al. showed that BCG promoted myelopoiesis at the expense of lymphopoiesis, and altered the transcriptional profiles of HSC and HPC (79). Mitroulis et al. demonstrated that administration of fungal cell wall component β-glucan to mice led to expansion of myeloid progenitor cells, metabolic adaptions in glycolysis and cholesterol biosynthesis (80). In both studies, the trained immunity-induced myelopoiesis contributed to protection against M. tuberculosis infection or chemotherapy-induced DNA damage and cell death (79, 80). Similarly, Christ et al. revealed that a cholesterol-rich diet had persistent effects on the myeloid progenitor cells, which were epigenetically and transcriptional reprogrammed. The trained myeloid progenitor cells maintained these phenotypes for a long period of time. The shift back to normal chow diet did not change the augmented pro-inflammatory immune responses in macrophages, which were supposedly the offspring of trained myeloid progenitor cells (81).

Though it is still not fully understood, the training of the HSC and HPC could be through the TLRs expressed on these cells. Long-term HSC and HPC subsets express TLR2 and TLR4, and thus can directly respond to the agonists to drive their differentiation toward myeloid cells (82). *In vitro* and *in vivo* stimulation of HSC/HPC with Pam3CSK4 or LPS led to myeloid differentiation (82–84). Moreover, direct exposure of human and mouse HSC/HPC to TLR1/2 agonist Pam3CSK4 led to the generation of macrophages that produced lower levels of inflammatory cytokines, and reactive oxygen species (85). Further studies demonstrated that the duration and the doses of the stimulation also played an important role to determine the nature of the responses. Short-term *in vivo* treatment with Pam3CSK4 led to a tolerance phenotype of *ex vivo* HSC/HPC-derived macrophages, whereas extended stimulation resulted in a trained phenotype. On the other hand, during the early stage of candidiasis infection, HSC/HPC differentiated to trained macrophages, while during the high candidiasis burden stage, HSC/HPC-derived macrophages was tolerized. Pam3CSK4-induced protection against candidiasis infection was abolished after HSC/HPC depletion, suggesting that the trained immunity memory was induced and maintained in the HSC/HPC population (86).

To develop a mucosal HIV/SIV vaccine, TLR2/6 agonist FSL-1 and MVA have been used as an adjuvant/delivery vector (26, 32). It is interesting to investigate the functionality of the monocyte/macrophages differentiated from FSL-1-primed HSC/HPC, especially when the signaling pathways through TLR1/2 and TLR2/6 had opposite roles to either silence or boost the immune responses (85).

Some long-lived tissue macrophages can maintain trained immunity memory. Respiratory viral infection induced trained immunity in alveolar macrophages, which mediated protection against bacterial infection with *Streptococcus pneumoniae*. The induction and maintenance of the memory was independent of monocyte or bone marrow progenitors (87).

Innate-like lymphocytes, which are present mainly in the mucosal surfaces and skin, also demonstrated immune memory (88). For example, Aspergillus protease-exposed Innate-lymphoid cell types 2 (ILC2) had a more vigorous cytokine production upon the re-challenge with papain (89). Moreover, NK and NK-like cells, as well as γδT cells from BCG-vaccinated individuals, produced more IFNγ (90). NKG2C^+^ NK cells have been reported to recognize HCMV-encoded UL40 peptides and to control the expansion and differentiation of adaptive NKG2C+ NK cells (91). NK cell mediated trained immunity memory has been recently reviewed (92), and will not be further discussed here.

Non-hematopoietic cells, like epithelial cells, can have memory to a previous contact with pathogen compounds (93). Pre-exposure of respiratory epithelia cells to Pseudomonas aeruginosa flagellin reduced or exacerbated inflammatory responses to a second non-related pathogen or LPS stimulation. By using histone acetyltransferase and methyltransferase inhibitors, the authors demonstrated that this was through epigenetic modifications.

## Other Factor-Induced Trained Immunity

To add an additional level of the complexity, other factors such as western diet (81, 94, 95), insulin (96), and microbiome, which have the potential to induce trained immunity, might also influence vaccine efficacy. To correctly interpret the immune correlates of protection for HIV/SIV vaccines, the contribution of trained immunity induced by these host factors needs to be taken into consideration.

Western-type diets (WDs) often include high calorically rich food that is lacking fiber, vitamins, and minerals. Long-term consumption of WDs can promote the activation of the immune system. While immune activation is the most important parameter to predict the susceptibility to HIV infection, recent studies also showed that WDs can alter *in vivo* LPS responses, and thus induce or alter trained innate immunity in monocyte/macrophages via NLRP3 (81, 95).

It has been known that trained immunity can be induced by endogenous metabolic products such as oxidized low-density lipoprotein (oxLDL), glucose glycylation end products, or fatty acids. For example, brief exposure of human monocytes to oxidized low-density lipoprotein (oxLDL) resulted in increased production of proinflammatory cytokines upon re-stimulation (97). This has been accomplished through trimethylation of lysine 4 at histone 3 (H3K4me3) in promoter regions of TNFα, IL-6, IL-18, the Matrix Metalloproteinase genes MMP2, MMP9, and the scavenger receptor CD36. Further study identified that ROS production, which was dependent on the AKT/mTOR signaling pathway, controlled the oxLDL-induced trained innate immunity phenotype (98).

Similarly, sustained activation of the AKT/mTOR signaling pathway leads to glycolysis in insulin resistance. Hyperglycemia facilitated sustained NF-κB gene activity due to increased H3K4 and reduced H3K9 methylation (99). Basal mTORC1 activity in the insulin-resistant macrophages was high. The macrophages showed an M2-like phenotype, and reduced their responses to LPS (100). Dietary changes also impact the gut microbiome, which has been shown to be associated with immune activation as well as HIV/SIV vaccine efficacy and susceptibility to infection (26, 101). In this context, western diet, or insulin signaling/insulin resistance signals have been viewed as modulators of trained immunity (98, 100, 102). Overall, various populations around the world might have potentially different responses to vaccines as they have different genetic backgrounds, diets, and microbiomes, which can affect innate and trained immunity.

## Harnessing the Power of Trained Immunity for AIDS Vaccine Development

The delivery modality for HIV vaccines is critical for vaccine efficacy. A large variety of delivery vectors and adjuvants have been used in AIDS vaccine development in either macaque models or human clinical trials. Among them, a large proportion demonstrated the ability to induce trained innate immunity, which means that they can impact the vaccine efficacy in a much longer time frame than we have thought before. Tables 1, 2 summarize the delivery vectors and adjuvants that have shown evidence (Table 1) or have the potential (Table 2) to induce trained immunity in experimental HIV/AIDS vaccines. Moreover, the hosts might have pre-induced trained immunity by factors such as diet, exposure history to other pathogens, and metabolic disorders. From these data one can speculate that trained innate immunity opens a new window for future HIV vaccine development: to design a novel HIV vaccine that combines the classical adaptive immunity as well as the protective trained immunity to mediate better protection than either one alone.

**Table 1:** The vector/adjuvant combinations found to induce trained immunity in experimental HIV/AIDS vaccines.

**Delivery vectors and adjuvants**	**Induced innate/trained immunity**	**Target cells involved**	**Associations with challenge outcomes**	**Minimum duration**	**Viral-specific adaptive immunity correlated**	**References**
MVA/TLR agonists/IL-15	APOBEC3G	mDC	Inverse correlation with set-point SIVmac251 viral loads	7 weeks	T cell-responses	(32)
ALVAC/DNA-SIV/alum	Hypoxia and the inflammasome	CD14+CD16-monocytes	Correlation with decreased risk of SIVmac251 acquisition	4 weeks	Anti-env antibody responses	(25, 27)
MVA/TLR agonists/IL-15/mLT	TNFα, IL-6, and MIP1α	Myeloid cells	Correlation with reduced viral Gag expression and *in vivo* viral acquisition	7 weeks	No T & antibody responses	(26)

**Table 2:** Adjuvants or vectors that have the potential to induce trained immunity in experimental HIV/AIDS vaccines.

**Delivery vectors and adjuvants**	**Induced innate immunity**	**Sample**	**Associations with**	**Measurement time**	**References**
TLR7/8 and 9 agonists	CXCL10	Plasma		Peak at 24 h, return to base level 1 week later	(34)
TLR7/8 agonist:R848		DC	Increased T cell responses		(35)
TLR4 and TLR7/8 agonists	Transcriptional profiling similar to live attenuated yellow fever vaccine	PBMC/monocyte	Anti-env antibody responses	24–96 h post immunization	(36)
ALVAC/MVA/NYVAC	Proinflammatory and antiviral cytokine/chemokine	Serum/PBMC		0–14 days post immunization	(28)

The concept of incorporation of protective trained immunity in HIV vaccine development is intriguing; however, it is far from being fully understood. Trained immunity induced by different stimuli or vaccinations might have these limitations: intermediate duration, non-specificity, limited local tissue distribution, and potential adverse effects such as potential enhancement of HIV/SIV viral acquisition. To harness the power of trained immunity in HIV/SIV vaccine development, we need to solve the following problems: (1) Practical assays to measure trained immunity; (2) Reliable methods/reagents to induce the two types of trained immunity, namely training and tolerance; (3) Long-term maintenance of the memory; (4) Identification of the protective trained immunity associated with HIV/SIV vaccine efficacy. Further studies to dissect whether sex bias plays any roles in trained immunity induction are also needed. Overall, long-lasting protective trained immunity provides new opportunities for innovative HIV vaccine design.

## Author Contributions

YS and JB designed the theme and topic and finalized the manuscript. YS drafted the manuscript. YS and JB drew the figure.

### Conflict of Interest

The authors declare that the research was conducted in the absence of any commercial or financial relationships that could be construed as a potential conflict of interest.
